# Gold nanoparticles grown inside carbon nanotubes: synthesis and electrical transport measurements

**DOI:** 10.1186/1556-276X-9-207

**Published:** 2014-05-03

**Authors:** Rodrigo A Segura, Claudia Contreras, Ricardo Henriquez, Patricio Häberle, José Javier S Acuña, Alvaro Adrian, Pedro Alvarez, Samuel A Hevia

**Affiliations:** 1 Instituto de Química y Bioquímica, Facultad de Ciencias, Universidad de Valparaíso, Av. Gran Bretaña 1111, Valparaíso 2340000, Chile; 2 Departamento de Física, Universidad Técnica Federico Santa María, Avenida España 1680, Valparaíso 2390123, Chile; 3 Centro de Ciências Naturais e Humanas, Universidade Federal do ABC, Rua Santa Adélia 166, Santo André, Sao Paulo 09210-170, Brazil; 4 Instituto de Física, Facultad de Física, Pontificia Universidad Católica de Chile, Vicuña Mackena 4860, Santiago 6904411, Chile

**Keywords:** Carbon nanotubes, Au-CNT hybrid, Electric transport, Gas sensing, 81.07.-b, 81.15.Gh, 81.07.De

## Abstract

The hybrid structures composed of gold nanoparticles and carbon nanotubes were prepared using porous alumina membranes as templates. Carbon nanotubes were synthesized inside the pores of these templates by the non-catalytic decomposition of acetylene. The inner cavity of the supported tubes was used as nanoreactors to grow gold particles by impregnation with a gold salt, followed by a calcination-reduction process. The samples were characterized by transmission electron microscopy and X-ray energy dispersion spectroscopy techniques. The resulting hybrid products are mainly encapsulated gold nanoparticles with different shapes and dimensions depending on the concentration of the gold precursor and the impregnation procedure. In order to understand the electronic transport mechanisms in these nanostructures, their conductance was measured as a function of temperature. The samples exhibit a ‘non-metallic’ temperature dependence where the dominant electron transport mechanism is 1D hopping. Depending on the impregnation procedure, the inclusion of gold nanoparticles inside the CNTs can introduce significant changes in the structure of the tubes and the mechanisms for electronic transport. The electrical resistance of these hybrid structures was monitored under different gas atmospheres at ambient pressure. Using this hybrid nanostructures, small amounts of acetylene and hydrogen were detected with an increased sensibility compared with pristine carbon nanotubes. Although the sensitivity of these hybrid nanostructures is rather low compared to alternative sensing elements, their response is remarkably fast under changing gas atmospheres.

## Background

Currently, the use of nanostructured templates or moulds has become a preferred way to build ordered structures organized over areas of hundreds of square micrometer in size. By depositing/casting the desired materials inside the templates, large arrays can be made efficiently and economically [1]. One of the simplest and most widely used materials for this purpose is opaline. It consists of spheres of glass, minerals, or plastic stacked in close-packed arrays. These arrays can either be produced naturally or artificially by induced self-assembly, for instance, by capillary forces [2]. Another method is through the use of polymer stamps. They are fabricated by casting on lithographically generated rigid moulds [3] or made using self-assembled copolymers deposited on flat substrates [4,5]. Another strategy to generate the template material is the use of anodized aluminum oxide membranes (AAOs). This type of membrane is usually prepared by the anodization of aluminum foils or thin films to obtain a honeycomb arrangement of pores perpendicular to the exposed surface [6-8]. This material has been used to build metal-insulator-metal nanocapacitor arrays for energy storage [9] and also to design highly specific and sensitive detectors for molecules of biological origin such as troponin, a protein marker for individuals with a higher risk of acute myocardial infarction [10].

Carbon nanotubes (CNTs) can be considered as an alternative nanoscale material with multiple applications in electronic and biological detection devices [11,12]. AAO membranes have been widely used to prepare CNTs using metal nanoparticles (MNPs) at the bottom of the pores and decomposing different carbon sources, at elevated temperatures [13-17]. In these cases, the MNPs catalyze the cracking of the gaseous hydrocarbons and also incorporate C atoms into their structures. The subsequent precipitation of a tubular structure happens once NPs have reached C supersaturation [18]. The diameter of the resulting CNTs is directly linked to the nanoparticle size [16] and synthesis temperature. Within certain limits, their lengths correlate well with the synthesis time [17].

Another approach to synthesize CNTs with AAO templates is the temperature-activated polycondensation of alkenes or alkyne derivatives. In this process, hydrocarbon units polymerize to form multiwall graphitic sheets, which follow the shape of the AAO membrane. The physical dimensions of the resulting products are determined by the shape of the pores. After the synthesis process is completed, the alumina mould can be dissolved and the CNTs released from its matrix. Using this method, it is then possible to prepare straight, segmented, and also branched CNTs but with a crystalline structure poorer than those grown by catalysis [19-22].

Several groups have successfully synthesized hybrid nanostructures composed of gold nanoparticles (AuNPs) attached to the outer surface of CNTs. They have mostly used covalent linkage through bifunctional molecules [23-25], while others have prepared hybrids only by taking advantage of the intermolecular interaction between the ligand molecules, usually long carbonated molecular chains bound to the AuNP surface and attached to the CNTs side walls [26-28]. Other metals have also been used to synthesize hybrids with CNTs. For example, AgNPs have been electrocrystallized onto functional MWCNT surfaces [29]. Magnetic iron [30], cobalt [31], and nickel [32] NPs have also been linked to CNTs to form hybrids structures. The use of these hybrids in magnetic storage as well as in nuclear magnetic resonance as contrast agents for imaging and diagnosis has been considered [33]. Other metals such as Pd [34], Pt [35], Rh [36], and Ru [37] have also been incorporated into CNTs mainly with the purpose of using them as catalysts or gas sensors.

Despite the large number of contributions regarding the synthesis of carbon nanotube-metal nanoparticle hybrid systems, only a few authors report the selective synthesis of metal nanocrystals inside CNTs. Using CVD, our group has synthesized CNTs by decomposition of acetylene on self-supported and silicon-supported AAO membranes [38]. These nanotubes are open at both extremes, if the membrane is self-supported and the barrier layer has been removed. Since the tubes' outside walls are initially completely covered by the AAO template, we can very easily access selectively the inside of the tubes by molecules or metal precursors in liquid solutions, while the outside wall remains free of any molecules or particles. We have used this principle to synthesize palladium and titanium dioxide nanostructures inside nanotubes [38]. Other authors have used closely related procedures to obtain platinum, nickel hydroxide, iron, and permalloy nanostructures [39-43].

In this report we have employed AAO membranes to synthesize supported CNTs arrays without the need to use metal catalysts. Taking advantage of the protection provided by the nanotubes by the hollow alumina cylinders, we have used these CNTs as nanoreactors to grow gold nanostructures selectively inside them. The nanotubes can subsequently be extracted from the AAO template to obtain hybrid peapod-like Au-CNT composites. Since our interest is evaluating the collective behavior of these hybrid nanostructures, interdigitated electrodes have been used to measure the conductance temperature dependence. Additionally, changes in the electrical resistance of these structures were verified under different atmospheric conditions in order to test the use of the new material as active elements in sensor devices.

## Methods

### Synthesis of CNTs and Au-CNT hybrid nanostructures

For the CNT synthesis, the catalytic decomposition of acetylene was carried out in a chemical vapor deposition apparatus (CVD), consisting of a horizontal tube furnace and a set of gas flow lines [44]. In a typical synthesis, performed at atmospheric pressure, a piece of alumina membrane (approximately 2 × 5 cm^2^) was heated at a rate of 20°C/min under an O_2_ stream (100 sccm) until reaching the desired synthesis temperature, (650°C). Then, O_2_ was replaced by Ar (100 sccm), and the system was kept under these conditions for 5 min. Acetylene (25 sccm) was later added for 10 min into the furnace. The hydrocarbon decomposes and the CNTs grow inside the porous AAO substrate to produce at the end a CNT-AAO composite. The sample generated by this procedure was labeled as CNT_(AAO/650°C).

For the Au-CNT hybrid synthesis, the CNT-AAO composite membranes were impregnated with a HAuCl_4_/2-propanol solution by dip-coating or drop-casting. Both methods were used in order to introduce quite different amounts of gold inside the CNTs. In the dip-coating procedure, a piece of membrane was completely immersed in a diluted gold solution (0.001 M) for 24 h. This sample was labeled as Au-CNT-A. To prepare a sample by drop-casting, 40 μL of a concentrated gold solution (1 M) was directly dropped on each side of approximately 1 × 1 cm^2^ piece of the CNT-AAO membrane. This sample was labeled as Au-CNT-B.

After impregnation, the pieces of membrane were placed in a tube furnace for calcination-reduction process. First, the membranes were dried at 150°C in an Ar stream (100 sccm) for 30 min. Then an O_2_/Ar mixture was added into the furnace and the temperature was raised up to 350°C for 1 h. Oxygen was later replaced by hydrogen (100 sccm), and the temperature was increased again up to 450°C for 1 h. The system was then cooled down to room temperature (RT) in an Ar flow.

### Purification and characterization of CNTs and Au-CNT hybrid nanostructures

To release the CNTs (with or without AuNPs), the membranes are immersed in a 5% aqueous NaOH solution for 24 h. This procedure dissolves the AAO. In addition, if ultrasonic dispersion is used (15 min at the beginning, 15 min after 12 h, and 15 min at the end of the 24-h period), the dissolution of the aluminas occur, since they have never been exposed to temperatures beyond the hardening phase transition. The CNTs and hybrids were purified by using a repetitive centrifugation process (three times), decanting the supernatant and using deionized H_2_O and 2-propanol to disperse them. The samples were subsequently dried at 150°C for 1 h in Ar.

Conventional transmission electron microscopy (TEM) and high-resolution TEM measurements were performed on the purified samples. For this purpose, small amounts of the purified and dried products were dispersed in 2-propanol in an ultrasonic bath (5 min). A drop of the dispersed sample was left to dry out over commercial holey carbon-coated Cu grids. Bright field micrographs were taken using a JEOL JEM 1200EX (JEOL Ltd., Tokyo, Japan) operating at 120 kV acceleration voltage, with a point resolution of approximately 4 Å. For high-resolution transmission electron microscopy (HRTEM) measurements, we used a JEOL JEM 2100 operated at 200 kV, with a point-to-point resolution of approximately 0.19 Å and equipped with an energy dispersive X-ray spectrometer (EDS) detector (Noran Instrument System, Middleton, WI, USA). The micrographs were captured using a CCD camera Gatan MSC 794 (Gatan Inc., Pleasanton, CA, USA). During the EDS measurements, a nanometer probe was used (approximately 10 nm in diameter) allowing the qualitative identification of both Au and C in the samples. Scanning electron microscopy (SEM) was also used to characterize CNTs and the Au-CNT films. SEM analysis was carried out using a LEO SEM model 1420VP (Carl Zeiss AG, Oberkochen, Germany; Leica Microsystems, Heerbrugg, Switzerland) operated between 10 and 20 kV. Raman spectroscopy was performed using a LabRam010 spectrometer (Horiba, Kyoto, Japan) with a 633-nm laser excitation.

### Transport measurements as a function of temperature

A 10-K closed cycle refrigerator system, from Janis Research Company (Wilmington, MA, USA), was used together with a Keithley electrometer model 6517B (Keithley Instruments Inc., Cleveland, OH, USA) in order to measure the current-voltage (*I*-*V*) curves as a function of temperature. The *I*-*V* curves were recorded in the absence of light and in high vacuum environment (<10^−6^ Torr). A drop of CNTs and Au-CNTs dispersions (2-propanol) was deposited onto interdigitated microelectrodes (IME) composed of platinum fingers (5 μm thickness × 15 μm gap) embedded in a ceramic chip. The resistance of IME-deposited CNTs and Au-CNTs is several orders of magnitude larger than the total resistance of the wires and electrodes; therefore, the errors introduced by using a two-probe measurement are negligible in this case. The conductance at zero bias voltage was obtained from the *I*-*V* curves while the temperature was ranged from 10 to 300 K.

### Room temperature transport measurements in different atmospheres

The electrical resistance of the CNT-covered IME chips was measured at room temperature in the presence of different gas mixtures. The IME chips were loaded into a vacuum chamber fitted with inlets for different gases. The concentration of the gases in each test is described below. The resistance was measured using a Keithley 6487 picoammeter. Some samples were measured with alternating current (AC), and lock-in amplifiers were used to acquire the voltages. The results of these measurements indicate that the changes in resistance are indeed dominated by the CNTs' response.

## Results and discussion

As already mentioned, for the synthesis of gold nanostructures inside CNT, a solution of HAuCl_4_ in 2-propanol was used to impregnate the CNT-AAO membranes. Drop-casting and dip-coating were both applied to impregnate the chloroauric solution in the membranes. After impregnation, the CNT-AAO membranes were calcinated (350°C) in an O_2_/Ar mixture and reduced (450°C) in a H_2_/Ar atmosphere. The alumina template was finally removed with a NaOH solution, leaving behind nanotubes filled with gold nanoparticles.

**Figure 1:**
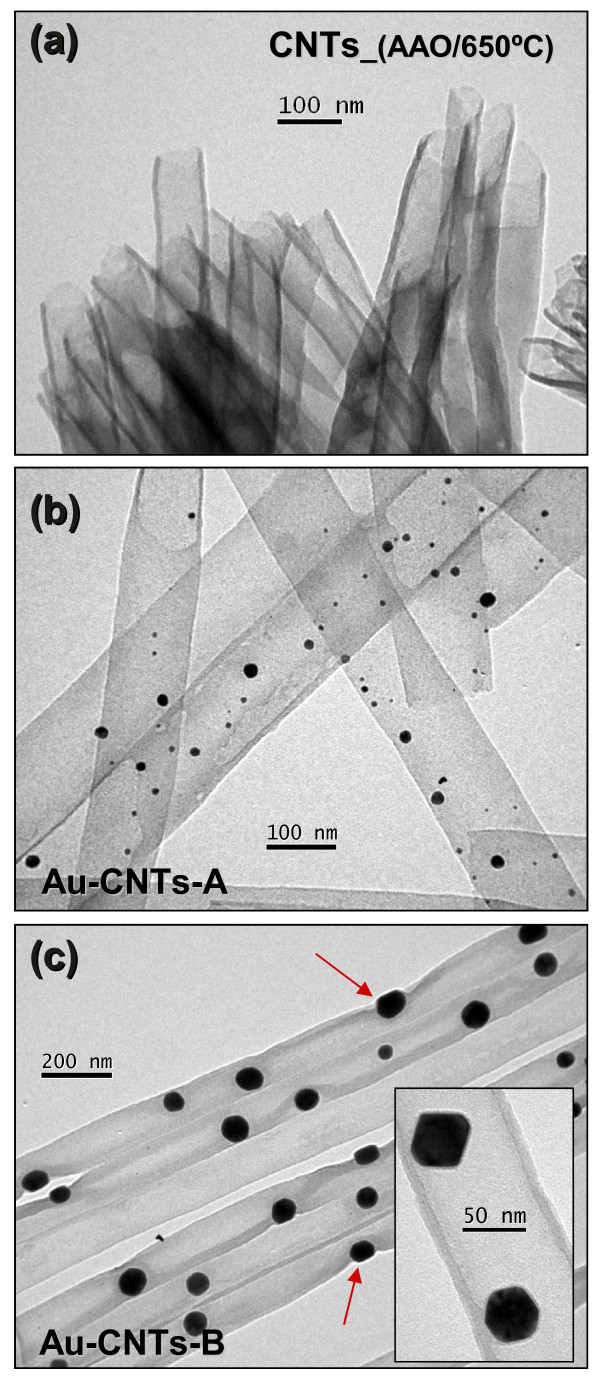
**TEM images of pure CNTs and Au-CNT hybrids. (a)** Pure CNTs prepared using the AAO template. **(b)** Au-CNT hybrids prepared by dip-coating method. **(c)** Au-CNT hybrids prepared by drop-casting. Important deformations are indicated by red arrows.

Figure 1 shows TEM images of the synthesized CNTs and the products obtained by reducing gold ions inside the nanotubes after the dissolution of the AAO membrane. Figure 1a shows a TEM micrograph of CNTs_(AAO/650°C) grown by decomposition of acetylene for 10 min. These CNTs exhibit a uniform diameter and uniform wall thickness with both ends open. As explained in our previous report, it is possible to control the wall thickness, hence the inner diameter of CNTs, by varying the exposure time to the hydrocarbon source [38]. In this contribution, we have used a 10-min synthesis time, which means the wall thickness is close to 7 nm. Figure 1b shows the Au-CNT hybrid nanostructures prepared by dip-coating method. In this case the ionic concentration in the CNTs' cavities is rather low (1 mM); hence, small gold nanoparticles were formed (2- to 10-nm mean diameter). Figure 1c shows the Au-CNT hybrid nanostructures prepared by the drop-coating method. In this case the nanoparticles have grown to a size close to 40 nm with evident facets in their geometrical structure, suggesting the formation of nanocrystals, as shown in the insert of Figure 1c. In this latter case, the gold ions were introduced by dropping a concentrated gold solution (1 M) directly onto the membrane. Larger agglomerates of gold precursor salt can be formed inside the tubes after a drying process, implying that larger nanoparticles can be formed after the calcination-reduction process; nevertheless, the maximum size of these agglomerates is determined by the inner diameter of the tube.

**Figure 2:**
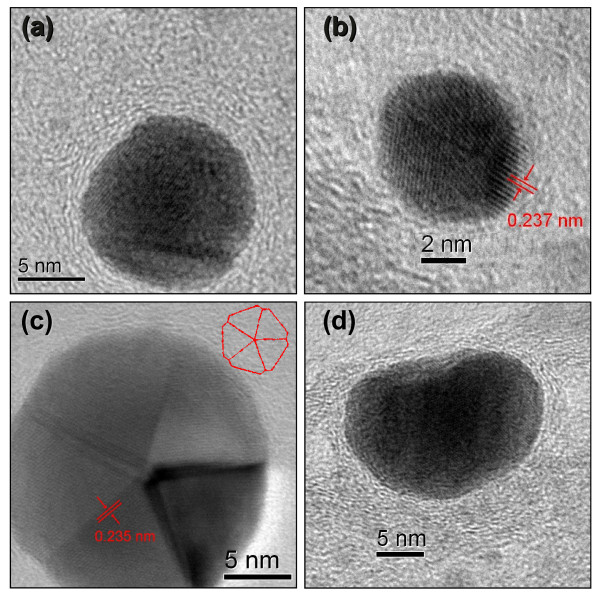
**HRTEM images of the hybrid nanostructures prepared by dip-coating (Au-CNT-A). (a-d)** Individual gold nanoparticles. **(a)** An onion-like carbon shell surrounding the AuNP. **(b, c)** The interplanar spacing, consistent with Au fcc, is highlight with red lines. The insert in **(c)** shows the shape of a decahedral object to allow comparison with the HRTEM image.

**Figure 3:**
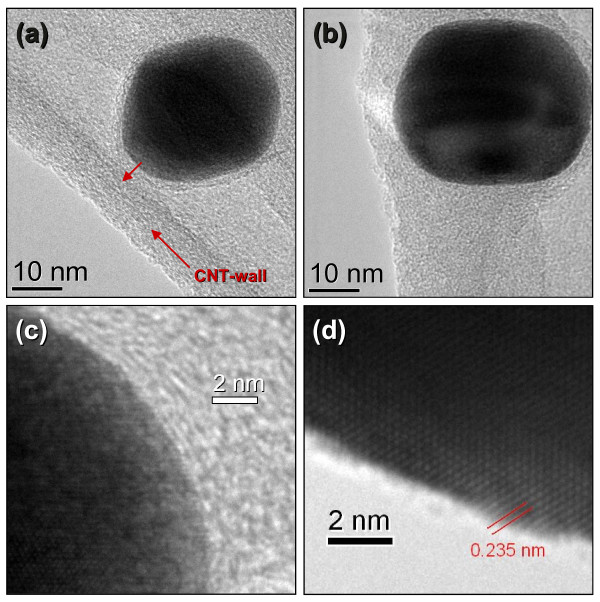
**HRTEM images of the hybrid nanostructures prepared by drop-casting (Au-CNT-B). (a-c)** The surrounding C shell and the AuNP-CNT interface can be observed. **(d)** Interlayer spacing of 0.235 nm is consistent with fcc (111) planes in Au.

Figures 2 and 3 show a set of HRTEM images for the hybrid nanostructures prepared by dip-coating (Au-CNT-A) and drop-casting (Au-CNT-B), respectively. In Figure 2, Au-CNT-A, it is possible to note that the nanoparticles acquire different sizes and shapes. A detailed examination revealed that these Au nanoparticles have indeed a face-centered cubic structure and dominant facets consistent with the (111) orientation of the crystal planes (2.35 Å interlayer spacing) [45]. Particularly, Figure 2c exhibits a fivefold twinned structure suggesting a decahedral shape [46,47]. In this last figure, we have inserted a view of a decahedral polyhedron to compare similarities with the NPs shapes in the HRTEM image. From Figures 2d and 3a,b, it is possible to verify that the AuNPs are attached to the inner wall of the nanotubes. These AuNPs are surrounded by a C onion-like shells, well attached to the CNT inner walls, as it has been verified previously [48]. These NPs, grown inside CNTs, can acquire the surrounding carbon layers by a relatively low-temperature activation process. Figure 3d shows an improved view of the structural order of the nanocrystals. In the same figure, the interlayer spacing of the encapsulated AuNPs has been highlighted, and again the (111) crystal plane is the dominant facet orientation.

From these images (Figures 1, 2, 3), it is then clear that gold nanostructures can be grown selectively inside the CNTs and attached to the inner walls. In this particular synthesis procedure, ions have the unique possibility of diffusing inside the CNTs through the open ends. After a calcination-reduction process, the gold salt agglomerates into zerovalent gold nanostructures inside the nanotubes. Our results indicate the lateral extent of the particles can be either limited by concentration of the Au precursors or by the tube's inner diameter when this concentration is high enough. We have also noted that during the formation of larger nanoparticle (Au-CNT-B), part of the CNT wall shrinks around it, causing important deformations as we indicated by arrows in the Figure 1c. In some cases, those particles appear to be outside the tubes, but closer observations indicate they are actually encapsulated by the CNT wall. In addition, the alumina is only removed after the nanostructures are formed, making very unlikely that the particles could be bound to the CNTs' external wall.

Lee et al. have reported the synthesis of nanotubes decorated with gold nanoparticles also by using AAO templates [49]. In this report, they have first prepared the AuNPs inside the AAO pores by impregnation of a gold dissolution and a thermal treatment. Then, they impregnate the Au-loaded AAO membrane with sucrose and subsequently a carbonization process was done in order to obtain bamboo-like carbon nanotubes filled with AuNPs. Their results show a scarce homogeneity in the physical distribution along the tube with a relatively wide particle size distribution.

**Figure 4:**
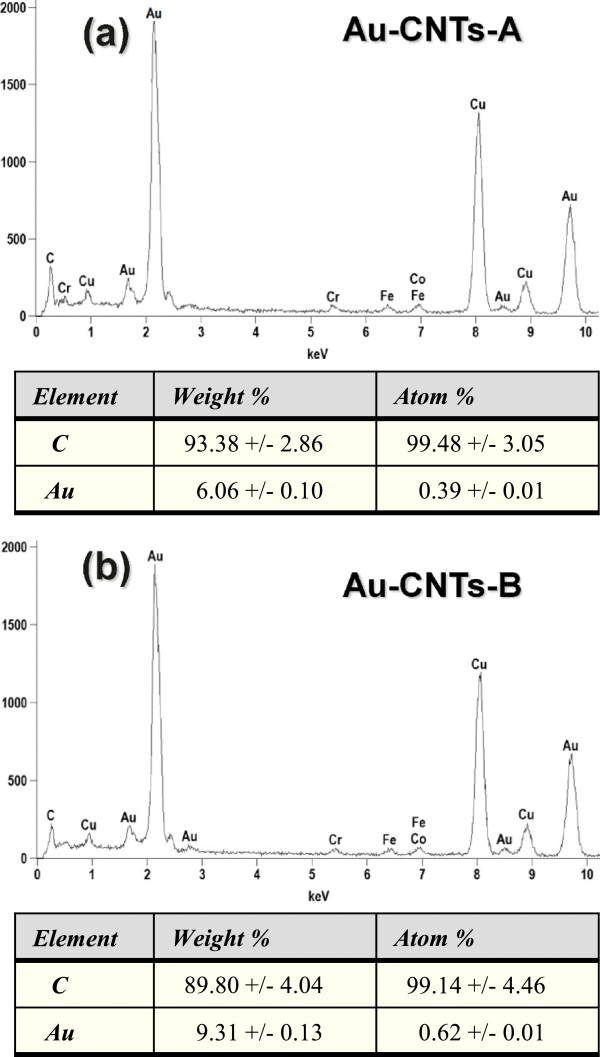
EDS analysis of the hybrid nanostructures prepared by (a) dip-coating and (b) drop-casting.

In order to corroborate the presence of gold in these hybrid structures, we have performed energy dispersive X-ray analysis with a 200-kV electron beam. Figure 4 shows typical EDS spectra for the samples prepared by dip-coating Figure 4a and drop-casting Figure 4b. Also, this figure displays tables with the weight and atomic percentage (%) for carbon and gold atoms in the hybrid samples. Even though the EDS analysis is a semi-quantitative method, it provides a clear confirmation that gold has been incorporated to the CNTs. Since the EDS signal from small nanoparticles is very low, the detection time for these NPs was increased; this explains the emergence of a copper signal, probably from the copper grid used to support the samples. Other elements such as iron and cobalt (due to TEM sample holders) have also been detected.

**Figure 5:**
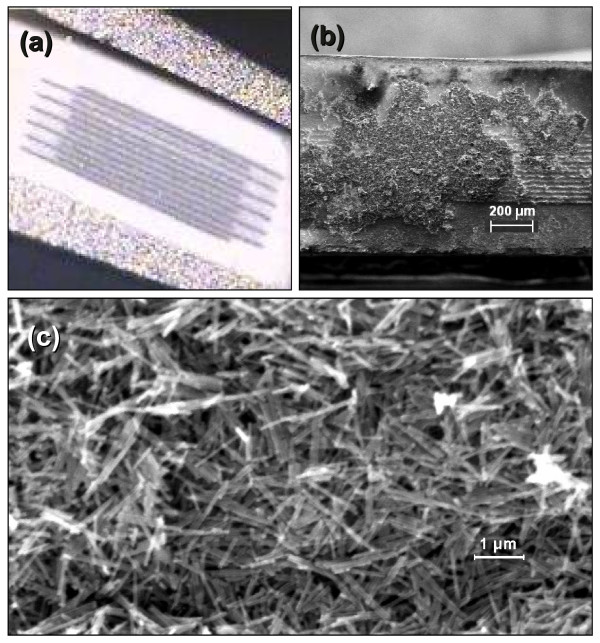
**Images of the IME chip and Au-CNT samples deposited over IME chip. (a)** Optical image of the IME chip. **(b, c)** Representative SEM images of Au-CNT samples deposited over IME chip.

To explore the electronic transport mechanisms and properties of these hybrid nanostructures, after being released, they were deposited on IME chips. Figure 5a shows an optical image of the IME chip. Figure 5b,c shows a typical SEM image of an IME chip with CNTs. In all the samples considered in this study, the CNTs and Au-CNTs hybrids were randomly oriented on the surface, forming a network of tubes between the electrodes.

**Figure 6:**
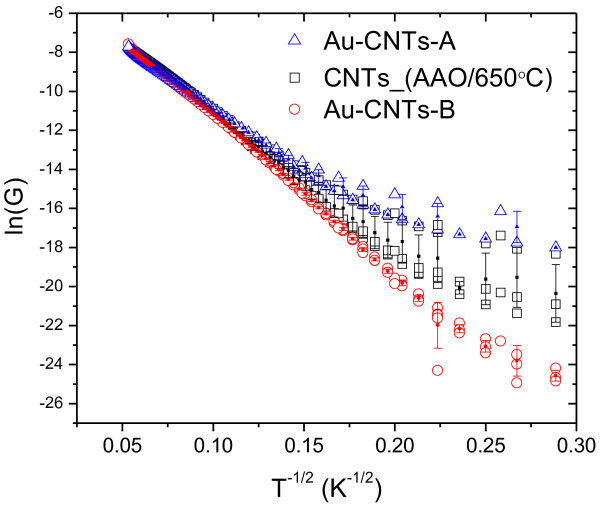
**Plots of ln(*G*) for the samples CNTs_(AAO/650°C), Au-CNTs-A, and Au-CNTs-B as a function of *T***^**−1/2**^**.** In addition to the measured data (open symbols), illustrative error bars have been included for each sample.

The first electrical measurement was oriented to obtain the temperature dependence of the sample conductance (*G*), at zero bias voltage, in high vacuum conditions, from 10 to 300 K. The conductance as a function of temperature for sample CNTs_(AAO/650°C), Au-CNTs-A, and Au-CNTs-B exhibit a non-metallic temperature dependence. Their conductivity can be explained using the variable range hopping (VRH) model in which charge carriers move by phonon-assisted hopping between localized states [50]. Therefore, the conductance at zero electric field can be obtained by Mott's law [51] as follows:

(1) $$G=G_{0}\exp{\left(-T_{0}/T\right)}^{1/\left(d+1\right)},$$

where *d* is the dimensionality and *T*_0_ *= α*^*3*^/*k*_B_*n*(*E*_f_) (the characteristic activation temperature). The parameter *α* is related to the decay of the localized electronic wave function, *n*(*E*_f_) is the density of localized one-electron states at the Fermi level, and *k*_B_, the Boltzmann constant. The best fit obtained for our data was for *d* = 1, consistent with a dominant 1D electronic transport mechanism in our samples. Figure 6 shows a plot of the natural logarithm of *G* as a function of *T*^−1/2^; the experimental data shows a linear dependence for almost the complete temperature range. By fitting the function in Equation 1, with *d* = 1, to the average data curve from sample CNTs_(AAO/650°C), a value of *T*_0_ *≈* 4.4 × 10^3^ K is obtained. For samples CNTs-A and Au-CNTs-B, the values of *T*_0_ from the fit of the average data were ≈ 4.4 × 10^3^ K and ≈ 5.0 × 10^3^ K, respectively. These results are in agreement with Wang et al.'s report [52], in which a 1D dependence within the VRH model is found for CNTs prepared using alumina templates. Although the values obtained for *T*_0_ are similar in all three samples, the inclusion of gold nanoparticles implies a larger value for *T*_0_. This is consistent with the fact that forming the gold nanoparticles by drop-casting (*T*_0_ ≈ 5.0 × 10^3^ K) produces noticeable modifications to the tubular structure of the CNTs compared to those generated through dip-coating (*T*_0_ ≈ 4.4 × 10^3^ K). As an example, several locations in which these changes occur have been indicated by arrows in Figure 1c. Figure 6 indicates that the inclusion of gold nanoparticles by drop-casting modifies the electronic transport below 60 K (see the curve with red open circle markers in Figure 6). In this low temperature range, only sample Au-CNTs-B exhibit the 1D hopping process, while the other two show a residual metallic behavior, inferred from the tendency to display a constant conductance. In the case of sample Au-CNTs-B, the residual metallic behavior of the conductance is almost non-existent and the VRH model can be extended to very low temperatures to account for the observed behavior. This result is consistent with the fact that the walls of the Au-CNT-B tubes are completely distorted by the presence of AuNPs, as detected by TEM (Figure 1c), and causing the suppression of the metallic conduction.

**Figure 7:**
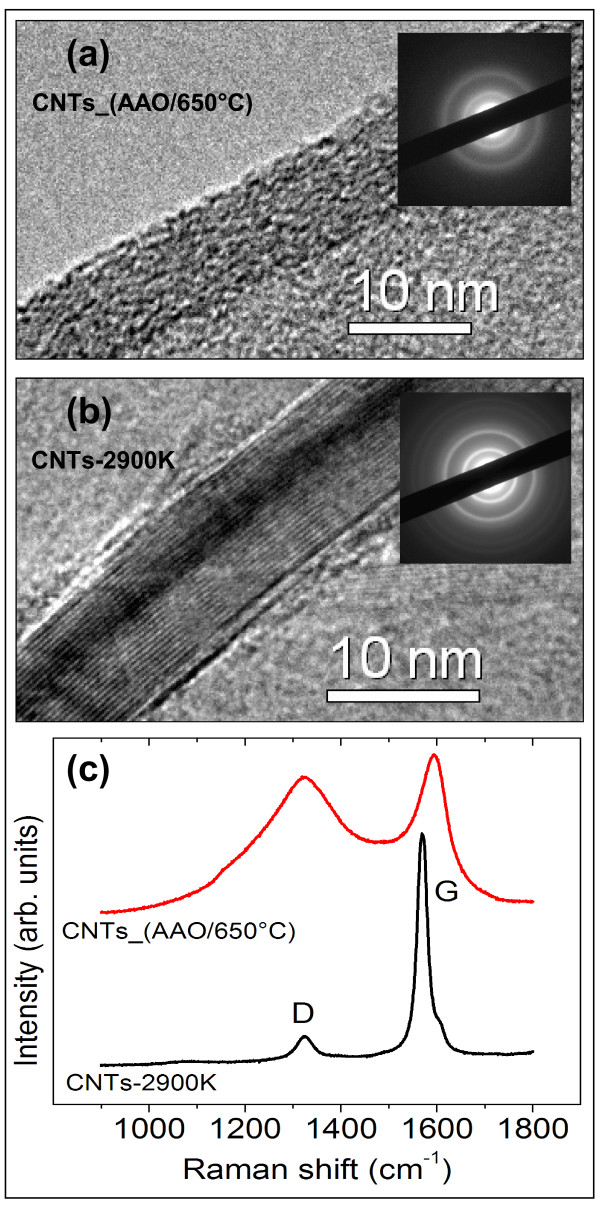
**HRTEM images, SAED patterns, and average Raman spectra from purified and annealed CNTs. (a, b)** Representative HRTEM micrographs of tube walls of the samples CNTs_(AAO/650°C) and CNTs-2900 K, respectively. The inserts in **(a)** and **(b)** are the diffraction patterns taken in the respective micrograph. **(c)** The average Raman spectra obtained from several measurements on different locations on the samples.

At this point, it is important to note that the transport measurements were performed using interdigitated microelectrodes, implying that conduction occurs through a mesh of CNTs between the electrode fingers (Figure 5c). Consequently, the interconnections between the CNTs need to be included in any model put forward to describe the conductance in this system. To verify this issue, we prepared an additional sample, labeled as CNTs-2900 K. It contains CNTs with a high degree of graphitization. These tubes were synthesized in the same way described in Section 2.1, but after their removal from the porous membrane, they were annealed in an inert atmosphere up to 2,900 K. Under this treatment, the tubes' shape and dimensions were conserved; however, the graphitization of their walls was dramatically increased. Figure 7a,b shows respectively HRTEM micrographs of the CNT's wall as grown and after the annealing treatment. The inserts in Figure 7a,b show the selected area electron diffraction (SAED) patterns of these samples, consistent with a higher degree of crystallinity of the CNTs after the thermal treatment. Figure 7c shows the average Raman spectra obtained from the corresponding samples. From the relative intensities of the *G* and *D* resonances, it is possible to conclude that the spectrum from CNTs-2900 K is consistent with a carbon sample with a high degree of graphitization [53-55], whereas the CNTs_(AAO/650°C) exhibits a structure with a considerable amount of amorphous carbon. Since the dominant electronic transport mechanism in amorphous carbon films [56] is based in a 3D hopping mechanism, it is not surprising that 1D hopping is the dominant electronic transport mechanism in sample CNTs_(AAO/650°C) as previously discussed.

**Figure 8:**
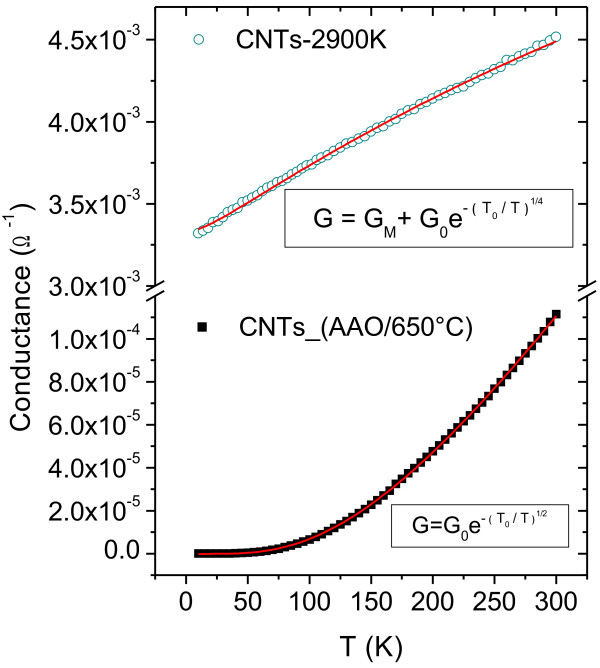
**Temperature dependence of the conductance for purified and annealed CNTs.** Temperature dependence of the conductance (*G*) measured at zero bias voltage for the samples CNTs-2900 K (green open circles) and CNTs_(AAO/650°C) (black squares). The red lines are the fit to the corresponding models; see text for further details.

Alternatively, the high degree of graphitization of the multiwalled tubes contained in the CNTs-2900 K sample, together with their large diameters, implies that these tubes should display a metallic behavior. Figure 8 shows the conductance's temperature dependence of samples CNTs-2900 K and CNTs_(AAO/650°C). The first remarkable discrepancy between both samples is the huge difference in their electrical conductance, both in magnitude and temperature dependence. Since both samples are built up from the same tubes, prior to annealing, this difference in conductance can be attributed mainly to modifications of the tubes' intrinsic electrical properties. Hence, the observed hopping transport mechanism in sample CNTs_(AAO/650°C) comes from the CNTs themselves and not only from the way they are dispersed on the substrate. On the other hand, the conductance in sample CNTs-2900 K increases to nearly linear as a function of temperature. This non-metallic temperature dependence could then be attributed to the junctions between CNTs. In order to explain the peculiar behavior of this sample, we can consider a 2-pathway model to describe its conductance [57]. One of them is dominated by the intrinsic metallic transport (*G*_M_) within the MWCNTs, while the other one is mainly due to the hopping mechanism (*G*_H_) between the tubes. Under this assumption, the conductance at low temperatures is given by the expression *G = G*_M_ *+ G*_H_, where *G*_M_ can be considered as roughly independent of temperature and *G*_H_ as having the same functional form as shown in Equation 1*.* The best fit for the free parameters (see Figure 8), considering 3D hopping, gave the following result: *G*_M_ ≈ 3.3 × 10^−3^ Ω^−1^, *G*_0_ ≈ 3.3 × 10^−2^ Ω^−1^ and *T*_0_ ≈ 3.8 × 10^4^ K. These values agree well with those obtained from exfoliated graphite in a similar experiment [57].

**Figure 9:**
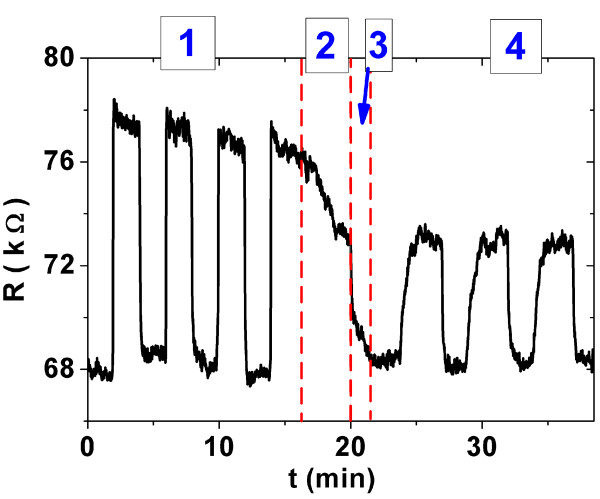
**Changes in resistance of CNT_(AAO/650°C) sample deposited on IME chip due to different environmental conditions.** In zone (1), vacuum/air cycles were performed (vacuum level is close 68 kΩ). In zone (2), air was replaced by argon. In zone (3), the chamber was pumped, and in zone (4), vacuum/Ar cycles were performed.

The electrical transport measurements were also performed under variable pressure conditions and room temperature. The purpose of this second set of measurements was to determine the effects of the different atmospheres in the electronic transport parameters of these samples. Figure 9 shows the sample resistance of CNTs_(AAO/650°C) subjected to several pressure cycles of the different gases. In zone (1), vacuum/air cycles were performed. In zone (2), air was replaced by argon. In zone (3), the chamber was pumped out. Zone (4) corresponds to the vacuum/Ar cycles.

**Figure 10:**
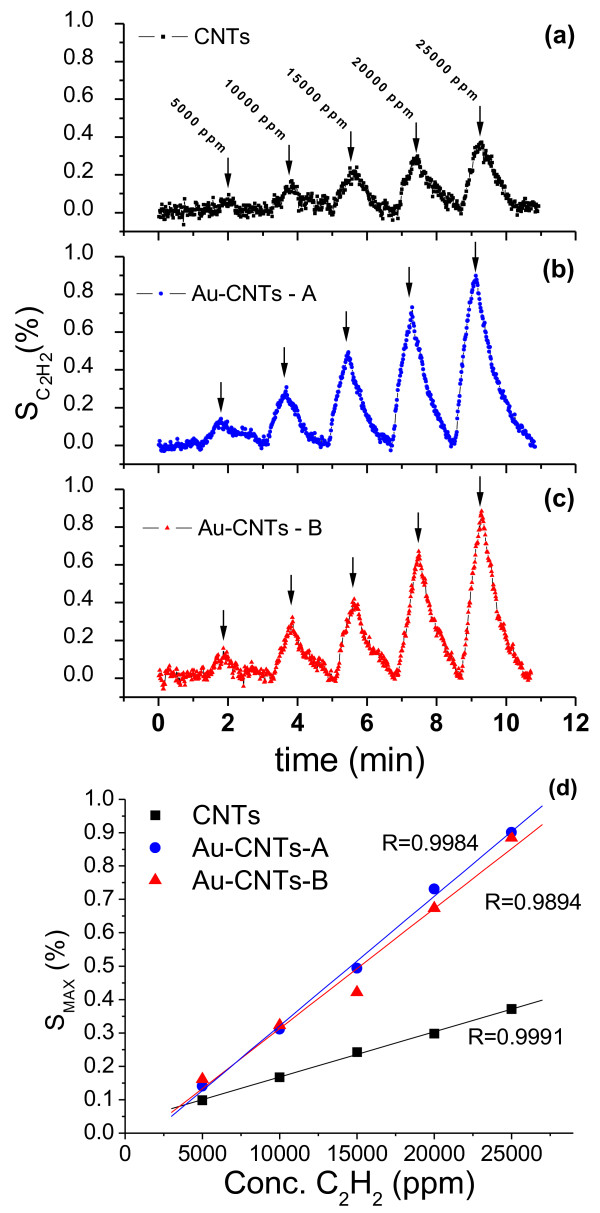
**Sensing response of CNT and Au-CNT samples towards the detection of acetylene (C**_**2**_**H**_**2**_**).** Response of pure CNTs **(a)** and response of hybrid Au-CNT samples prepared by dip-coating **(b)** and drop-casting **(c)**. Plot of the maximum sensitivity value for each peak as a function of C_2_H_2_ concentration **(d)**. The solid lines in **d** graph are linear fits to the corresponding data points.

The resistance changes observed between the different sampling zones suggest that these materials could be used as chemiresistor gas sensors. This concept has been verified by running several cycles of alternating gas mixtures. For example, cycles of Ar (100 sccm × 2 min) as baseline gas, followed by a mixture of Ar/C_2_H_2_ (×0.5 min) were considered. The mixture started with 2 sccm of C_2_H_2_ until it reached 10 sccm by increasing 2 sccm in each cycle while keeping constant the total gas flow at 100 sccm. These nominal amounts of acetylene in the incoming mixture have been transformed, taking into account the volume of the vessel used as detection chamber (close to 200 cc) and the amount of gas feed during the half minute, to actual concentration near the sensor surface. Consistently, the amounts of acetylene near the sensor were varied from 5,000 ppm, for 2 sccm nominal concentration to 25,000 ppm for 10 sccm. The electrical resistance of the chips was recorded as a function of time and later the data was transformed to ‘sensitivity’ defined as the variation of resistance due to the gas mixture (Δ*R* = *R*_*i*_-*R*_0_) normalized by the resistance of the baseline (*R*_0_, pure Ar in this case) in percentage, *S* (%) [58]. The resulting data of this experiment is presented in Figure 10. These plots show the sensitivity (%) of CNTs (Figure 10a), Au-CNT-A (Figure 10b), and Au-CNT-B (Figure 10c) for C_2_H_2_ detection. Apart from the final concentration of the analyte, there are several facts that can be extracted from Figure 10. First, the sensing material always detected acetylene in the working range at room temperature. Although pure CNTs are able to detect C_2_H_2_, with a maximum sensitivity of 0.37% for Au-CNT-(A and B), the maximum sensitivity value was close 0.90%. These numbers indicate a relatively high increase in the sensitivity to hydrocarbons for the gold-loaded CNTs. No significant differences were found between the dip-coated and the drop-casted samples. Another important fact is that sensitivity rises linearly with the analyte concentration for all samples which can be seen in the graph of Figure 10d. In this figure, we have plotted the maximum sensitivity as a function of acetylene concentration in parts per million, which is well described by a linear fit to the data. The *R* values of these fit are very close to 1. Within this linear range, these materials could be used for the determination of an unknown concentration of this particular gas. Additionally, these samples display a rapid response and recovery times to variations in the gas mixture.

Penza et al. have studied the sensing properties of CNTs decorated with gold particles [59]. They sputtered thin gold layers over thick CNT films with vacuum-evaporated Au-Cr leads. This report shows that the substantial improvements in the gas (NO_2_, NH_3_, and H_2_S) sensing properties of CNTs are indeed induced by gold. Their results are consistent with a high sensitivity at 200°C; nevertheless, this material, in most cases, has larger detection and recovery times.

**Figure 11:**
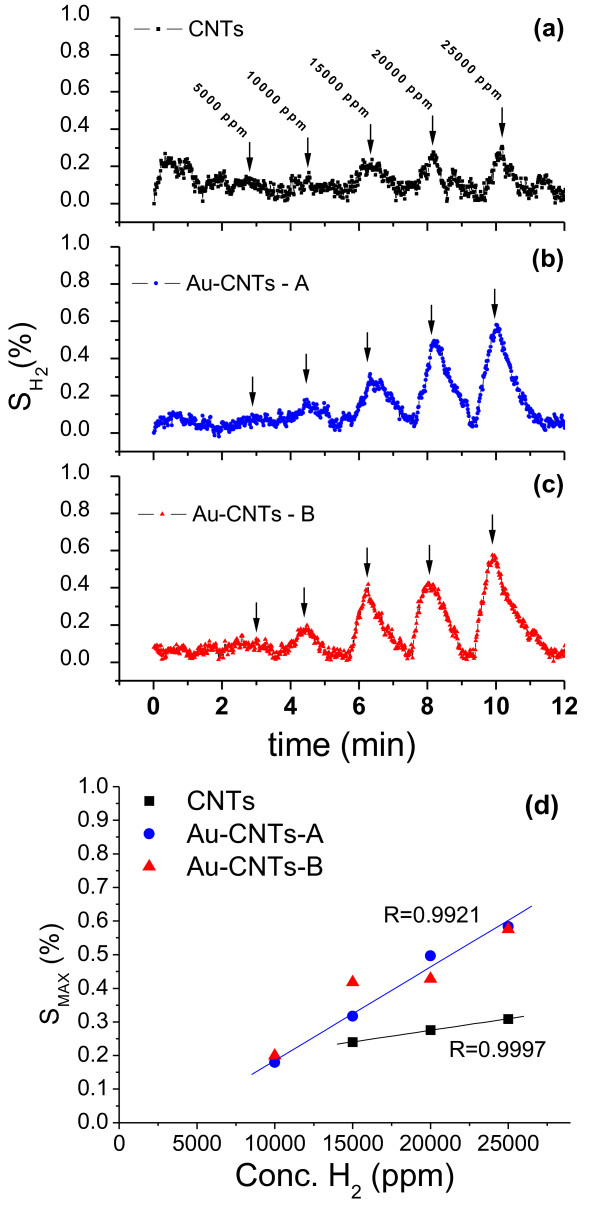
**Sensing responses of CNT and Au-CNT samples towards the detection of hydrogen (H**_**2**_**).** Behaviour of pure CNTs **(a)** and hybrid Au-CNT samples prepared by dip-coating **(b)** and drop-casting **(c)**. Maximum sensitivity value for each peak as a function of H_2_ concentration **(d)**. Solid line in **d** graph represents the linear fit to these data points.

In addition, we have performed a similar set of measurements using hydrogen as the analyte gas. The sensitivity (%) plots of H_2_ vs time for CNTs and Au-CNT hybrid samples are presented in Figure 11. All samples are less sensitive to H_2_ than acetylene. Pure CNTs display very little sensitivity. In the case of Au-CNT samples, no significant signal was detected for low H_2_ concentration (5,000 to 10,000 ppm), and the linearity of the signal with concentration is not as good as in the case of C_2_H_2_, (Figure 11d). Sadek et al. have electrocrystallized AuNPs on nitrogen-doped CNTs and use them in hydrogen detection [60]. In their sample, platinum metal leads were sputtered directly onto the film to improve the electrical contacts. The high sensitivity values obtained in this report could be explained as due to the large number of gold clusters interacting with hydrogen molecules and causing charge transfer to the CNT network. This charge transfer is expected to induce substantial modifications in the electrical transport parameters.

In both cases, CNT and Au-CNT hybrids, increased resistance under acetylene or hydrogen exposure has been detected. This behavior is typical of p-type semiconductors exposed to electron donor gases, since these species induce a reduction of the density of holes [59]. Particularly for Au-CNT hybrid nanostructures, the sensing mechanism could be explained by two process: (1) the adsorption of gases in the side walls of CNTs, with the simultaneous charge transfer between the target molecule and the CNT network; and (2) the gold nanoclusters could be producing a catalytic spillover effect, in which the electron donor gases are chemisorbed and their electrons transferred from the gold particles to the CNT, decreasing the conductivity of the p-type material. It is worth mentioning at this point that the presence of the AuNPs can modify the catalytic activity of the hybrids not only due to the presence of the particles themselves but also because of the structural changes they induce in the walls of the CNTs, thus modifying the intrinsic chemical affinity of the tubes.

The difference in sensitivity of the gold-modified CNTs in this report, compared to previous reports [59,60], could be due to the lower density of NPs used in the course of this study. This report indicates that hybrid materials formed by AuNPs, encapsulated by the CNTs, are useful as sensing elements; nevertheless, further characterizations are indeed required in order to incorporate them in practical devices.

## Conclusions

Through the procedures described in this report, we have indeed formed a nanoscale reactor with physical dimensions that can be designed by adjusting the synthesis procedure. These reactors are fairly uniform in diameter and while protected by AAOs, added particles, precursors, or molecules only can access the inside of the tubes. As a way to prove the effectiveness of this strategy, we have selectively located Au ions inside the tubes' cavities. Depending on the preparation conditions, the AuNPs can be made to evolve from small NPs, with diameter only dependent on the precursor concentration, to larger conglomerates with sizes that are fixed by the CNT's confinement. The alumina can be easily dissolved releasing the new CNT-particle hybrids. From the study of the conductance as a function of temperature, we found that the dominant transport mechanism in the CNTs_(AAO/650°C) and the Au-CNTs samples is the intra-tube 1D hopping. This is consistent with the fact the CNTs' walls contain a considerable fraction of amorphous carbon. Below 60 K, the inclusion of AuNPs inside the CNTs introduces changes in the electronic transport, in particular, with the method of the drop-casting, which modify the CNTs' walls.

We have also proved that random arrays of our Au-CNT-hybrid samples supported on IME chips are able to detect small amounts of a hydrocarbon gas as acetylene with a fast response and a fast recovery time. These sensors show a linear response with respect to gas concentration in the case of acetylene, whereas in the detection of hydrogen, they display a poorer sensitivity and linearity.

## Abbreviations

AAO: anodized aluminum oxide; AC: alternating current; CCD: charged coupled device; CNT: carbon nanotube; CVD: chemical vapor deposition; EDS: energy dispersive X-ray spectroscopy; HRTEM: high-resolution transmission electron microscopy; IME: interdigitated microelectrode; MNPs: metal nanoparticles; NPs: nanoparticles; SEM: scanning electron microscopy; TEM: transmission electron microscopy; VRH: variable range hopping.

## Competing interests

The authors declare that they have no competing interests.

## Authors' contributions

The work presented here was carried out in collaboration among all authors. RS and SH defined the research theme. CC, AA, and PA carried out the synthesis and transport experiments under the supervision of RS, RH, and SH. RS performed TEM measurements, JJSA, the HRTEM and EDS analysis, and SH, the SEM and Raman measurements. RS, SH, RH, JJSA, and PH have discussed all this results and RS, SH, and PH wrote the manuscript. All authors read and approved the final manuscript.
